# Nuances in the Evaluation of Chest Pain in Women

**DOI:** 10.1016/j.jaccas.2021.07.035

**Published:** 2021-12-01

**Authors:** Kelsey Vargas, Anne Messman, Phillip D. Levy

**Affiliations:** a Department of Emergency Medicine, Cleveland Clinic Foundation/Case Western Reserve University, Cleveland, Ohio, USA; b Department of Emergency Medicine, Wayne State University, Detroit, Michigan, USA; c Integrative Biosciences Center, Wayne State University, Detroit, Michigan, USA

**Keywords:** acute coronary syndrome, chest pain, myocardial infarction, women, ACS, acute coronary syndrome, AMI, acute myocardial infarction, CAD, coronary artery disease, HEART score, History, ECG, Age, Risk factors, Troponin score, MACE, major acute coronary event, MINOCA, myocardial infarction with nonobstructive coronary arteries, PCI, percutaneous coronary intervention, SCAD, spontaneous coronary artery dissection

## Abstract

Although chest pain is the most common presenting symptom for both men and women who ultimately receive diagnoses of acute coronary syndrome, there in are important differences in coronary artery disease pathophysiology that can affect patient care. Using a case-based approach, we provide insight into these and other important considerations that every clinician should think of when treating women with chest pain. (**Level of Difficulty: Intermediate.**)

## Case Presentation

A 45-year-old healthy woman presented to the emergency department for evaluation of chest pain and fatigue that had been present for 4 days but was now worsening. She was currently going through a divorce and had been experiencing more stress than usual. Her symptoms were triggered by feeling anxious or stressed, with spontaneous resolution soon afterward. However, the evening before presentation she began experiencing sharp, persistent chest pain that was not relieved with antacids or rest. Upon awakening this morning, the pain was still present, 5/10 intensity, and was associated with overwhelming fatigue. She did not describe any recent cough, fever, or chills. She had experienced some nausea but no vomiting or diarrhea. She had been eating less because of the nausea and the stress of her divorce.

**Learning Objectives**

• To educate readers on the unique aspects of chest pain presentation that occur in women.
• To describe causes of acute coronary syndrome that are more common among women, using a case of spontaneous coronary artery dissection to highlight distinctions.

Her vital signs on arrival were as follows: blood pressure 145/80 mm Hg, heart rate 85 beats/min, respiratory rate 18 breaths/min, temperature 37 °C, and pulse oximetry 99% on room air. Her body mass index was 27 kg/m^2^.

Physical examination showed a well-appearing woman without diaphoresis, normal respirations with clear lung sounds, and no cardiac murmurs or lower extremity edema. The results of abdominal and neurologic examinations were normal, as were her peripheral pulses.

**Figure 1:**
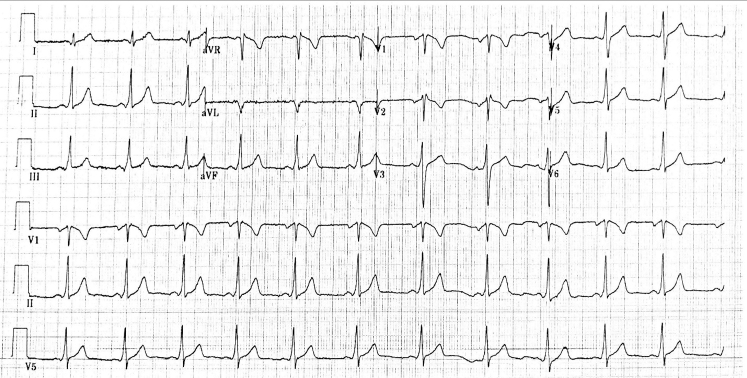
Initial Electrocardiogram

**Figure 2:**
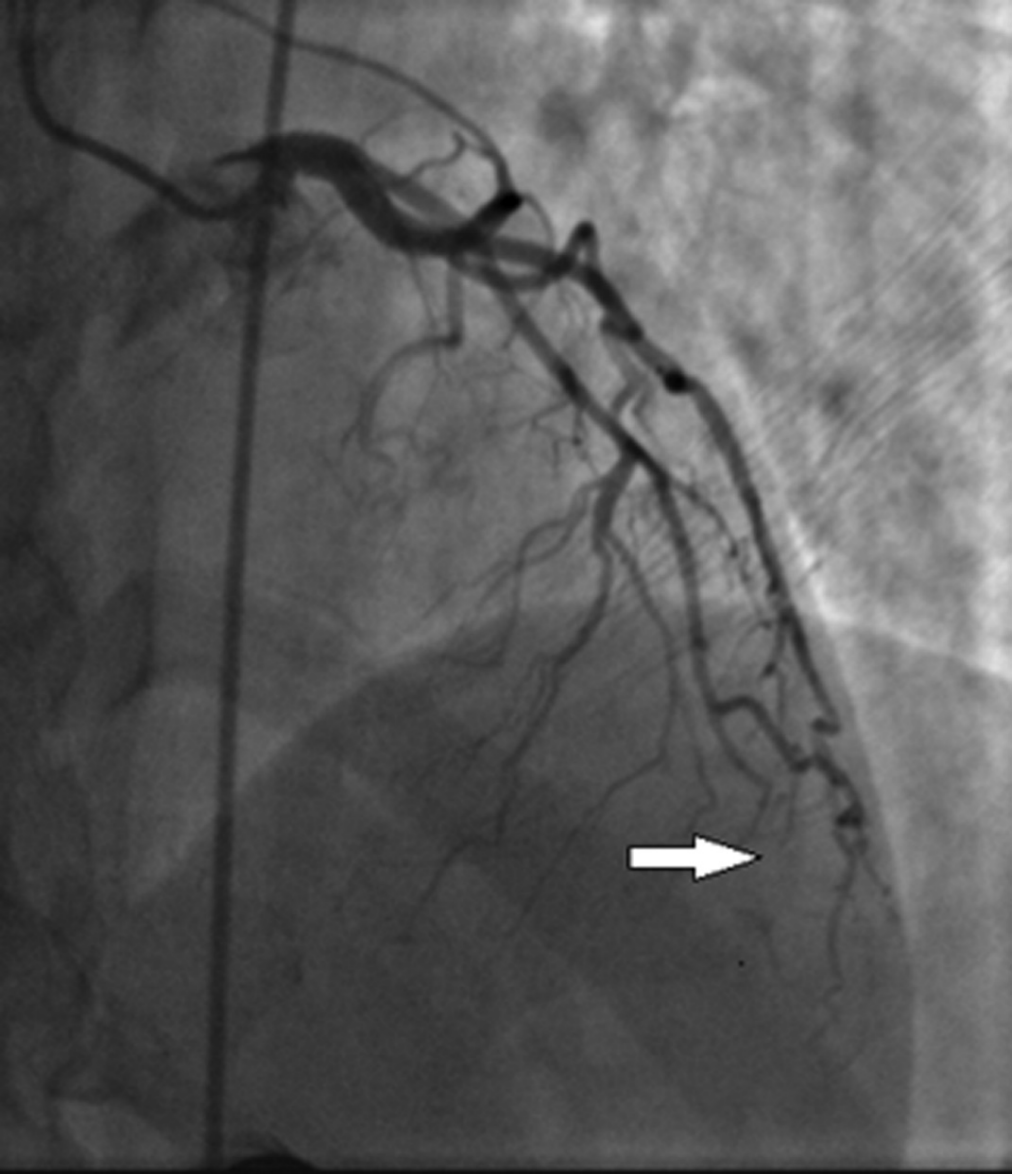
Coronary Angiography Demonstrating Dissection of the Distal Left Anterior Descending Artery Image provided by Nicole Pristera, MD, Department of Cardiovascular Medicine, Heart and Vascular Institute, Cleveland Clinic.

An electrocardiogram was obtained which showed an incomplete right bundle branch block with no evidence of ST-segment elevation or depression (Figure 1). Her initial laboratory test results were remarkable only for hypokalemia at 3.2 mmol/L (normal range 3.5-5.2 mmol/L); her initial high-sensitivity troponin I was detectable but below the 99th percentile. A chest radiograph was negative for any acute process. Repeated troponin determination at 2 hours was 20 ng/L. The cardiology service was called, and the patient was admitted for further testing. An echocardiogram showed a normal ejection fraction, with no wall motion abnormalities. She underwent a coronary angiogram the next day and was found to have a spontaneous coronary artery dissection (SCAD) (Figure 2, Video 1).

## Question 1

Do women with acute coronary syndrome (ACS) present differently from men?

### Answer 1

In both women and men with diagnoses of ACS, chest pain is the most common presenting symptom. One study showed that 93% of women presenting with ACS had chest discomfort or pain (including pressure and heaviness) (1). Among stable outpatients with suspected coronary artery disease, women more often characterized their pain as crushing, pressure, squeezing, or tightness than men (52.5% vs 46.2%; *P* < 0.001) (2). However, women are more likely than men to experience and identify associated symptoms as the primary symptom (3). Associated symptoms include unusual fatigue, shortness of breath, upper back pain, flulike symptoms, dizziness, generalized scared/anxious feeling, indigestion, and palpitations. Therefore, for women who present with any of the listed associated symptoms, a thorough review of systems, specifically inquiring about chest pain or chest discomfort, can help elucidate the cause of the presentation.

## Question 2

Does the pathophysiology of acute myocardial infarction (AMI) differ in men from that in women?

### Answer 2

Differences in plaque characteristics between men and women are thought to derive from the interaction between coronary pathologic features and biologic sex characteristics (3). Plaque rupture or type 1 is the most common cause of AMI in both men and women; however, plaque erosion is seen more frequently in women, which allows AMI to occur in the presence of nonobstructive coronary artery disease (CAD), also known as myocardial infarction with nonobstructive coronary arteries (MINOCA) (4,5). One study found that obstructive CAD was more often absent than present in Black women with ACS despite there being evidence of myocardial necrosis (6). Therefore, ensure discussion with the interventional cardiologist regarding specific concerns for SCAD in women.

Another rare cause of ACS is SCAD. Associated with high mortality, SCAD often exists in the absence of the well-known risk factors for ACS (7). SCAD has been associated with peripartum and postpartum status, oral contraceptive use, exercise, connective tissue disorders, and vasculitides. Among women, it was found to be the cause in up to 35% of patients with a mean age of 42 to 53 years, a major acute coronary event (MACE) rate of 47.4%, and a 10-year morality rate of 7.7%. High MACE and mortality rates require admission for further evaluation and treatment, which can be complicated with a potential for wire insertion into a false lumen, expansion and extension of dissection or hematoma, or need for greater than anticipated stent placement in the performance if percutaneous coronary intervention (PCI). Therefore, discussion with the cardiology service regarding specific concerns about SCAD in women who present with chest pain or associated symptoms in the absence of traditional risk factors can facilitate consideration of treatment options that may be otherwise delayed or overlooked.

## Question 3

Are women less likely to have CAD compared with men?

### Answer 3

There is a lower prevalence of atherosclerotic CAD in women than in men (3). Particularly low rates are seen in young women, and the prevalence increases with age, as expected. However, the relationship between sex and CAD is modified by other risk factors, making it imperative that the low pretest probability of ACS does not anchor the physician to a non-ACS diagnosis. Although there has been some concern that such scoring systems underrepresent women because of the greater likelihood of uncommon history or associated symptoms, a recent study found that women deemed to be at low risk by the HEART (History, ECG, Age, Risk factors, Troponin) score received guideline-concordant care more often than men, resulting in fewer hospitalizations and less noninvasive cardiac testing (8). Therefore, even though women are less likely to have CAD, sex should not be used as the sole criterion for risk stratification because the risk of ACS and CAD depends on multiple other factors.

## Question 4

Does psychosocial stress have an impact on the risk of AMI in women more than in men?

### Answer 4

The outcomes for AMI in women may be related to emotional stress. Young women with early-onset myocardial infarction have been found to have a disproportional burden of psychosocial risk factors, despite similar or more favorable risk factors in comparison with men of similar age (3). A recent study found that mental stress–induced AMI was more common in women aged 50 and younger than in age-matched men (9). However, the trend was not observed in ages 50 years and older. In addition to high levels of depression, young women with ACS have been found to have high rates of poverty and trauma exposure during childhood (10). A link between depression and ischemic heart disease has emerged and is now recognized as a prognostic factor after ACS (10). Therefore, emerging evidence shows a link between psychosocial factors and the development of ACS and ischemia.

## Question 5

How do racial and ethnic perceptions affect the evaluation of chest pain in women?

### Answer 5

Secondary to a range of factors, the prevalence of AMI is higher in Black women than in all other racial and ethnic groups (3). Young Black women have higher hospitalization rates for AMI than do young white women, as well as an increased incidence of comorbidities such as hypertension, chronic kidney disease, diabetes, and heart failure (3). Black, Hispanic, and American Indian populations have also been shown to present later to the hospital after the onset of symptoms, which affects the outcomes. Education on symptoms and outreach programs are seen less frequently in these populations, which could be contributing to later presentations. Last, there are documented disparities in PCI and coronary artery bypass grafting, as well as decreased referrals for coronary angiography and reperfusion. It is difficult to study the construct of these disparities; however, implicit bias may be a contributing factor. Increasing adherence to guidelines theoretically would reduce these disparities and increase the care for ACS patients of all backgrounds.

Using these 5 questions, we have highlighted factors related to presentation, perceptions, prevalence, pathophysiology, and psychological stress (Central Illustration) that should be considered whenever evaluating women with chest pain.

**Central Illustration:**
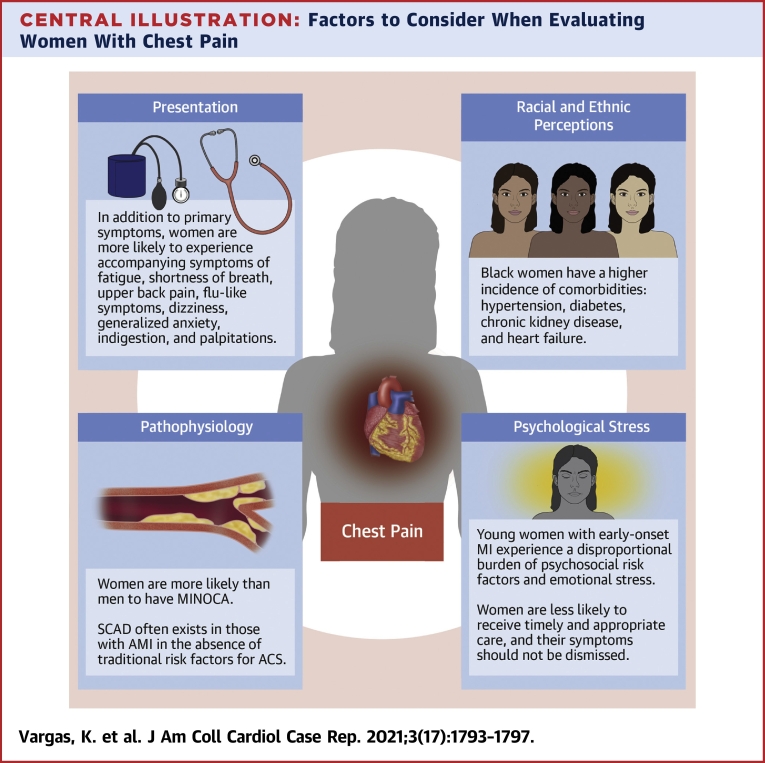
Factors to Consider When Evaluating Women With Chest Pain ACS = acute coronary syndrome; AMI = acute myocardial infarction; MI = myocardial infarction; MINOCA = myocardial infarction with nonobstructive coronary arteries; SCAD = spontaneous coronary artery dissection.

## Funding Support and Author Disclosures

Dr Levy has been a recipient of NHLBI (R01 HL146059 and R01 HL127215), NIH Admin (U24 NS100680), MDHHS (CDC 1815 and1817); MHEF (R-1907-144972); of research contracts from Pfizer and Novartis; and of consulting fees from BMS and AstraZeneca. All other authors have reported that they have no relationships relevant to the contents of this paper to disclose.

## Appendix

Supplemental Video 1 Coronary angiogram showing left anterior descending artery dissection. Image provided by Nicole Pristera, MD, Department of Cardiovascular Medicine, Heart and Vascular Institute, Cleveland Clinic.
